# The impact of anions on electrooxidation of perfluoroalkyl acids by porous Magnéli phase titanium suboxide anodes

**DOI:** 10.1371/journal.pone.0317696

**Published:** 2025-01-23

**Authors:** Yaye Wang, Yifei Wang, Shuping Dong, Qingguo Huang

**Affiliations:** 1 Jiangsu Provincial Academy of Environmental Science, State Environmental Protection Key Laboratory of Aquatic Ecosystem Health in the Middle and Lower Reaches of Yangtze River, Nanjing, People’s Republic of China; 2 Department of Crop and Soil Sciences, College of Agricultural and Environmental Sciences, University of Georgia, Griffin, Georgia, United States of America; Linköping University: Linkopings universitet, SWEDEN

## Abstract

Previous studies have indicated the great performance of electrooxidation (EO) to mineralize per- and polyfluoroalkyl substances (PFASs) in water, but different anions presented in wastewater may affect the implementation of EO treatment in field applications. This study invetigated EO treatment of perfluorooctane sulfonate (PFOS) and perfluorooctanoic acid (PFOA), two representative perfluoroalkyl acids (PFAAs), using porous Magnéli phase titanium suboxide anodes in electrolyte solutions with different anions present, including NO_3_^-^, SO_4_^2-^, CO_3_^2-^ and PO_4_^3-^. The experiment results indicate that CO_3_^2-^ enhanced PFAS degradation, while NO_3_^-^ suppressed the degradation reactions with its concentration higher than 10 mM. SO_4_^2-^ and PO_4_^3-^ exhibited less impact. Further studies with electrochemical characterizations and radical quenching experiments illustrate the mechanisms of how the anions may impact EO performance.

## Introduction

Per- and polyfluoroalkyl substances (PFASs) constitutes a large family of human-made chemicals that have developed for a few decades, comprising at least one perfluoroalkyl moiety,–C_n_F_2n_– [1, 2], with perfluorooctanoic acid (PFOA) and perfluorooctane sulfonate (PFOS) being the most well-known species. Due to extensive application and unregulated disposal, PFASs can be detected in almost all environment media, including air [3], soil [4–7], groundwater [8–10] and even drinking water [11, 12]. PFASs are thermally and chemically stable with low reactivity, leading to their global distribution and accumulation [11, 13]. However, PFASs are shown closely correlated with numerous adversary health effect, including increased liver enzymes and cholesterol [14, 15], decreased birth weight and fetal growth [16, 17], and even kidney and testicular cancer [18, 19]. Due to their extensively existence, environmental persistence and potential carcinogenicity and toxicity, PFASs raises public concern since early 2000s. US Environmental Protection Agency has recently announced the enforceable levels of 4.0 ppt for PFOA and PFOS individually in drinking water [20].

The stability of PFASs and their surfactant nature make them highly resistant to many treatment technologies, such as advanced oxidation processes (AOPs), because hydroxyl free radicals (HO^∙^) cannot effectively attack C-F bonds [21–23]. Numerous destructive technologies are being developed in order to fully mineralize PFASs, including photochemical oxidation [24, 25], sonochemical treatment [8, 26], plasma-based technology [27, 28], alkaline hydrothermal treatment [29, 30] and advanced reduction processes [31–33]. These destructive technologies are however still under development at different phases, suffering various limitations in terms of energy efficiency and demanding conditions. For example, the electrical energy necessary to reduce 90% PFOS(EE/O) for photochemical oxidation, plasma-based technology, and electron beam treatment were reported to be 122.22 kWh·m^-3^, 23.2 kWh·m^-3^ and 102–193 kWh·m^-3^, respectively [24, 27, 34]. Biodegradation was also shown effectiveness in PFAS removal [35–37], with up to 60% PFOA and PFOS removal reported for an enrichment culture of *Acidimicrobium* sp. strain A6 after 100 days of incubation, forming shorter-chain PFASs and fluoride ion as the intermediate products. 6:2 fluorotelomer sulfonate (6:2 FTS) was found to biotransform to PFPeA, PFHxA, and 5:3 Acid in aerobic sediments, while did not biodegrade under aerobic conditions.

Electrooxidation (EO) treatment is a promising wastewater treatment technology to destruct recalcitrant organic contaminants under ambient temperature and pressure [38–40]. EO occurs via anodic oxidation to degrade PFASs, primarily by direct electron transfer (DET) [41–43] with reactive oxygen species (ROS) also playing a role to facilitate the process [44, 45]. Both anode materials and water matrix may affect the performance of EO on PFASs degradation. The observed reaction rate constant normalized by geometric surface area of PFOS ($k_{SA}^{\prime }$) were 2.06×10^−5^ m•s^-1^, 8.48×10^−6^ m•s^-1^ and 8.40×10^−6^ m•s^-1^ on Ti/TiO_2_-NTs/Ag_2_O/PbO_2_, Ti/PbO_2_ and Ti/TiO_2_-NTs/PbO_2_ anodes, respectively, when the current density was 30 mA•cm^-2^ [46]. For BDD and Si/BDD anodes, the $k_{SA}^{\prime }$ of PFOS degradation were 2.19×10^−6^ m•s^-1^ and 2.80×10^−5^ m•s^-1^, when the current densities were 15 mA•cm^-2^ and 23.24 mA•cm^-2^, respectively [47, 48]. Porous Magnéli phase Ti_4_O_7_ material exhibits high conductivity and electrocatalytic reactivity, chemical and thermal stability, long performance life and high oxygen evolution potential (OEP), thus making a promising candidate for electrochemical oxidation of PFASs as anode [49–51]. Since Ti_4_O_7_ anodes have porous structure, more electroactive sites are available for electrochemical reaction to take place. 99.5% PFOA and 93.1% PFOS were degraded on Ti_4_O_7_ anodes after 180 min EO reaction at 2.7 V vs. SHE in spiked water with 0.5 mM PFOA and 0.1 mM PFOS as initial concentrations using 20 mM NaClO_4_ as supporting electrolytes [52]. However, natural waters commonly have different anions present, such as CO_3_^2-^, SO_4_^2-^, NO_3_^-^ and PO_4_^3-^, which may compete with PFASs for the active sites on anode, while their effects on PFASs degradation during EO treatment have not been systematically examined.

Higher temperature was proved to favor the degradation of PFOA, with the removal rate increased from 83.3% to 90.7% after 30 min of EO treatment at 10 mA∙cm^-2^ when the temperature increased from 10°C to 22°C [53]. However, the presence of other organic pollutants, such as trichloroethylene (TCE), apparently inhibited the degradation of PFOS [54]. There was still a lack of a systematic evaluation of the effect of different anions on PFAS degradation. This study aims to explore the effects of nitrate, sulfate, carbonate and phosphate ions on the degradation of PFOA and PFOS as model PFAS in water during EO with Ti_4_O_7_ anode. These anions are common in surface water and industrial wastewater. Spectroscopic and electrochemical characterizations were employed to elucidate the mechanisms underlying the effect of different anions. The results of this study provide useful information to guide the implementation of Ti_4_O_7_-based EO treatment for PFAS removal in contaminated waters or concentrated waste streams.

## Materials and methods

### Chemicals and reagents

The chemicals and reagents involved in this work were provided in the supporting information (S1 Text in S1 File) in detail.

### Anode fabrication and characterization

The process of Ti_4_O_7_ fabrication was described in our previous study [45]. In brief, the Ti_4_O_7_ powders were obtained by reducing TiO_2_ powder at 950°C in H_2_ atmosphere. The Ti_4_O_7_ powder was then pressed in the mold to form a green body. During this process, polyacrylamide/polyvinyl alcohol (95/5, m/m) was used as the binder to help molding. Finally the green body was further heated to 1350°C and maintained at 1350°C to form a bulk electrode. Vacuum is required during the whole sintering process. The dimension of the Ti_4_O_7_ anode used in this study was 70 mm in length, 45 mm in width, and 2.5 mm in thickness. The weight of the anode is 81.5 g.

The physical properties of the Ti_4_O_7_ anode were characterized by a number of techniques, such as XPert PRO MRD X-ray diffractometer (XRD) with CuKα1 radiation at 45kV/40mA (PAnalytical, Netherland) over the 2-Theta range of 10–80°. The morphology is observed by Hitachi SU-8230 Scanning electron microscopy (SEM) (Schaumburg, USA). MicroActive AutoPore V 9600 (Norcross, GA) was used to characterize the size and volume of pores in the anode. Electrochemical characterizations including anodic potential (AP) measurement, linear sweep voltammetry (LSV) and cyclic voltammetry (CV) were performed on a CHI-660E electrochemical workstation (Austin, TX) with a leak-free Ag/AgCl reference electrode (CH Instrument). All anodic potentials in the study have been corrected with internal resistance (iR) compension and reported against hydrogen electrode (SHE).

### Electrooxidation experiments

The experiments using spiked reaction solution were conducted in a self-designed acrylic batch reactor (6.50 cm×5.50 cm×5.70 cm) with the Ti_4_O_7_ plate as the anode. The volume of reaction solution is 100 mL throughout the study. 316 stainless steel plate was used as the cathode. The anode and cathode were in parallel placement and the distance between them was fixed at 2.5 cm. Constant current was supplied by a DC power source (Tacklife Inc, China). The initial concentration of PFOS and PFOA were 2.0 μM for each, and the supporting electrolytes is 100 mM NaClO_4_. Sodium salts, NaNO_3_, Na_2_SO_4_, Na_2_CO_3_ and Na_3_PO_4_, were added into the reaction solution at 1.0, 5.0, 10 or 20 mM to assess the effect of different anions on PFOS degradation. Duplicate 400 μL samples were collected at each predetermined time.

### Chemical analysis

400 μL methanol containing 80 ppb M8PFOS and M8PFOA were added to the each 400 μL aliquot, and then filtered through a 0.22 μm nylon. All samples were kept below 4°C and analyzed within 28 days of experiments. An ultra-performance liquid chromatography coupled with a triple-stage quadrupole mass spectrometer (ACQUITY UPLC-MS/MS, Xevo TQD, Waters Corp., USA) was used for PFAS separation and quantification. The gradient program of UPLC is listed in S1 Table in S1 File. The detailed MS parameters were summarized in S2 Text in S1 File. The standard of quality assurance and quality control was also included in S2 Text in S1 File. The MS transition and detection limit of PFOA/ PFOS are listed in S2 Table in S1 File. M8PFOA and M8PFOS were used as the isotope-label internal standard of PFOA and PFOS, respectively (S3 Table in S1 File).

## Results and discussion

### Anode characterization

**Fig 1:**
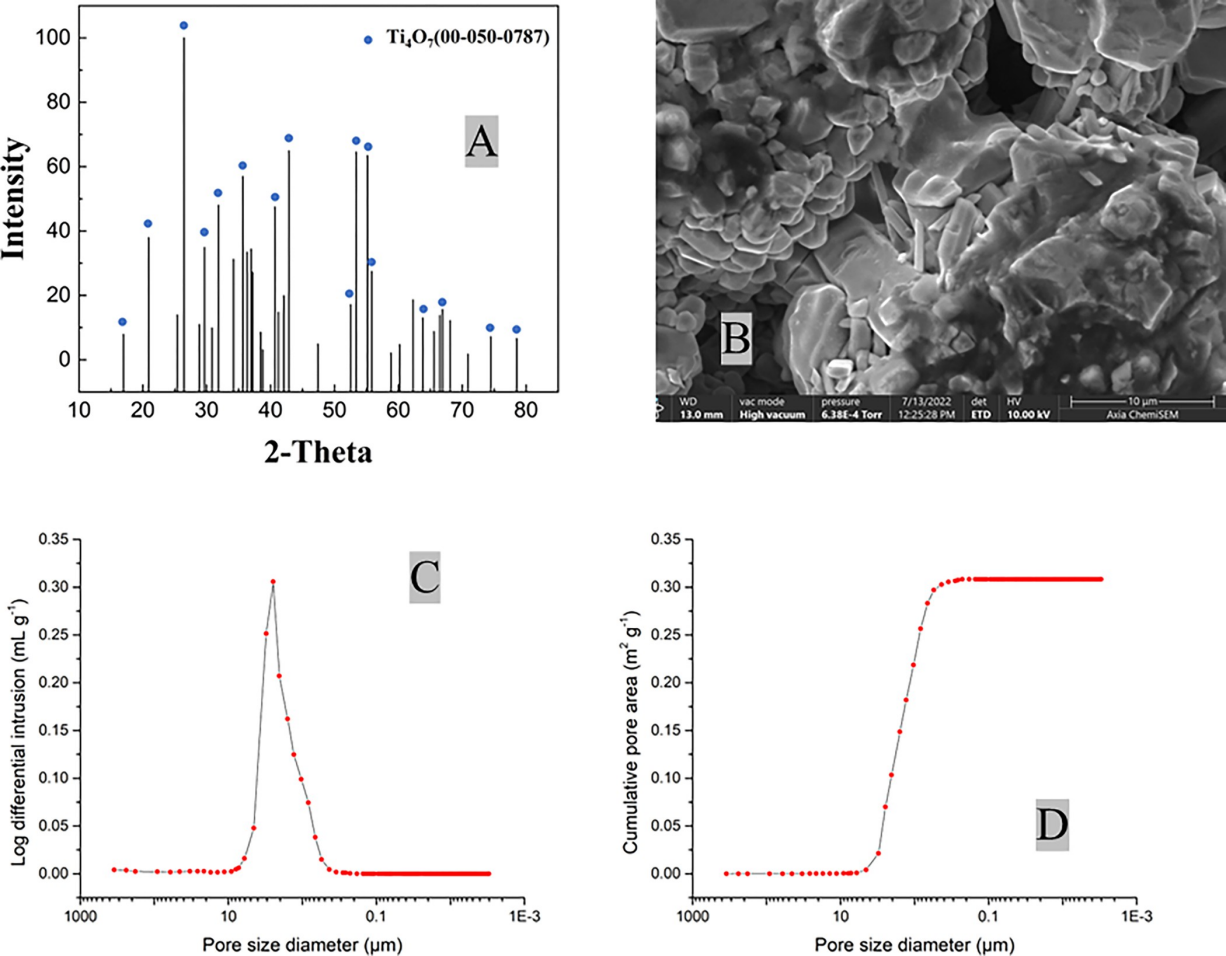
The XRD (A), SEM (B) and mercury intrusion porosimetry analysis on pore size distribution (C) and cumulative pore surface area (D) of the Ti_4_O_7_ anode. The blue dots represent the characteristic peaks of ICDD Ti4O7 (00-050-0787).

The main crystalline phase of anode was identified by XRD via matching characteristic peak of standard materials (Fig 1A). The XRD patterns of the Ti_4_O_7_ anode exhibits that it is composed of 74.4% Ti_4_O_7_, 18.7% Ti_5_O_9_ and 6.90% Ti_6_O_11_. The SEM result reflects that the Ti_4_O_7_ anode has a highly porous structure (Fig 1B).

The distribution of pore diameter of the Ti_4_O_7_ anode was shown in Fig 1C and 1D. The porosity was 21.6%, and the average pore diameter at 4 V∙A^-1^ was 2.6 μm. The total pore area was 1.29×10^−5^ cm^2^. However, since inner surface of porous anode cannot be accessible by electrolyte, the “outer” surface where the redox reaction can take place, also called effective electroactive surface area (EESA) can better depict the actual reaction sites on porous anode [55–57]. Voltametric method was used to measure EESA of Ti_4_O_7_ anode [58–60] (S3 Text in S1 File) in different supporting electrolyte solutions, and EESA of anode in different reaction solution are presented in Table 1, S4 Table and S1 Fig in S1 File. It appears that adding anions with increasing concentrations help to enhance EESA. This may be because higher electrolyte concentration compresses the diffusion layer at a charged interface, while a thinner diffusion layer results in a higher electroactive surface area [61]. Higher charge valences of different anions may also contribute to thinner diffusion layer [62].

**Table 1:** EESA of Ti_4_O_7_ anodes in different electrolyte solutions.

Electrolyte	EESA (cm^2^)
**100 mM H_3_PO_4_**	2035.71
**100 mM Na_3_PO_4_**	2016 .33
**100 mM NaClO_4_**	1927.00
**100 mM NaClO_4_+1 mM NaNO_3_**	1970.66
**100 mM NaClO_4_+5 mM NaNO_3_**	2053.36
**100 mM NaClO_4_+10 mM NaNO_3_**	2356.55
**100 mM NaClO_4_+20 mM NaNO_3_**	2447.65
**100 mM NaClO_4_+1 mM Na_2_SO_4_**	1993.34
**100 mM NaClO_4_+5 mM Na_2_SO_4_**	2110.56
**100 mM NaClO_4_+10 mM Na_2_SO_4_**	2287.86
**100 mM NaClO_4_+20 mM Na_2_SO_4_**	2395.38
**100 mM NaClO_4_+1 mM Na_3_PO_4_**	2264.62
**100 mM NaClO_4_+5 mM Na_3_PO_4_**	2484.64
**100 mM NaClO_4_+10 mM Na_3_PO_4_**	2636.31
**100 mM NaClO_4_+20 mM Na_3_PO_4_**	2730.26
**100 mM NaClO_4_+1 mM Na_2_CO_3_**	1942.86
**100 mM NaClO_4_+5 mM Na_2_CO_3_**	2221.25
**100 mM NaClO_4_+10 mM Na_2_CO_3_**	2238.04
**100 mM NaClO_4_+20 mM Na_2_CO_3_**	2427.57

**Fig 2:**
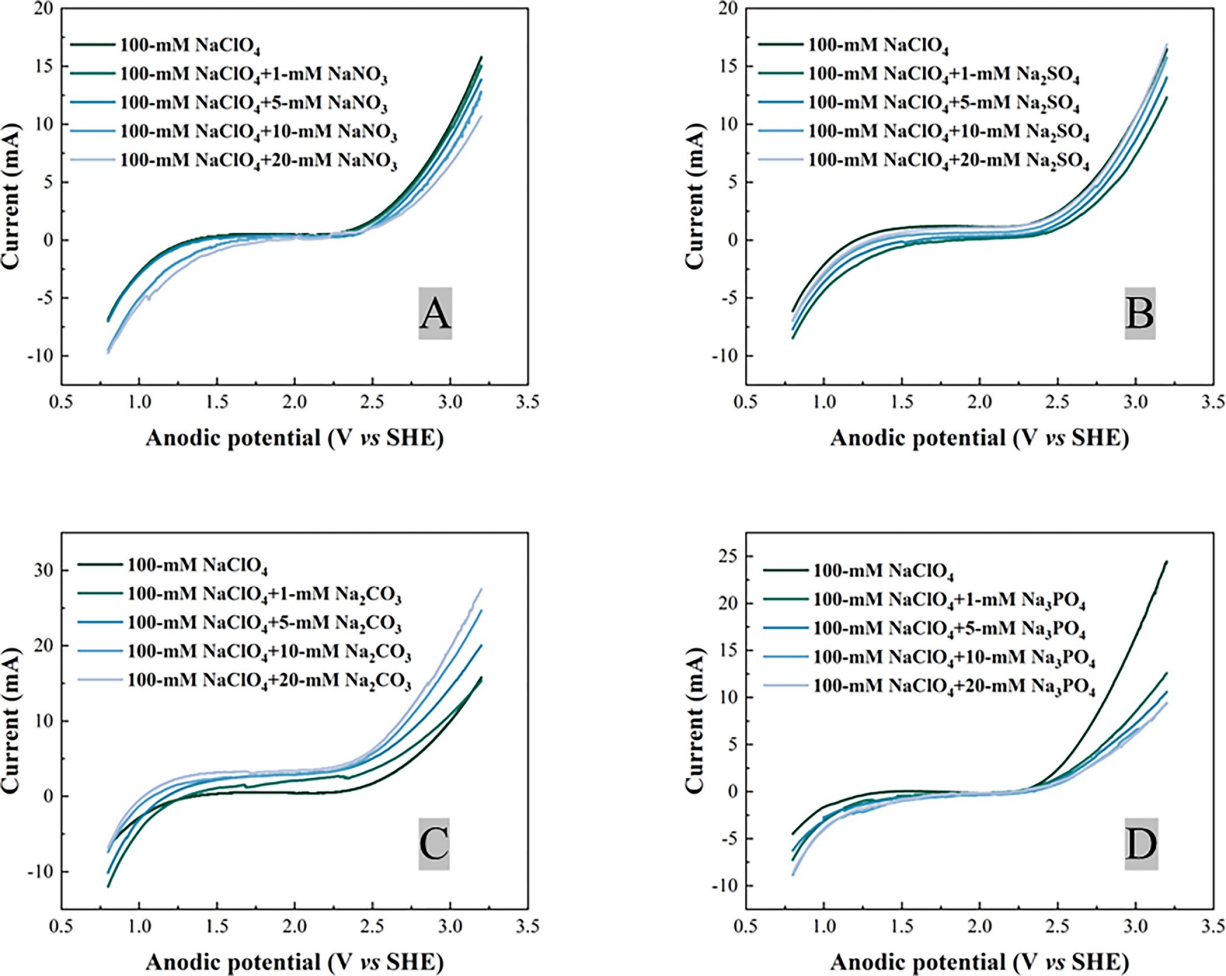
LSV results of the Ti_4_O_7_ anode in different electrolyte solutions at scan rate 50 mV·s^-1^: NO_3_^-^ (A), SO_4_2^-^ (B), CO_3_^2-^(C) and PO_4_^3-^ (D).

The LSV results of Ti_4_O_7_ anode in 100 mM NaClO_4_ solution with different concentration of different anions (1–20 mM) was shown in Fig 2. For NO_3_^-^, SO_4_^2-^ and PO_4_^3-^, the anodic current in reaction solution with only 100 mM NaClO_4_ was higher than that with other anions added, (Fig 2A, 2B and 2D). It suggests that electrolysis of water was hindered due to reactive sites of Ti_4_O_7_ anode has been taken up by these anions, leading to reduced generation of HO^∙^ on the anode surface [45, 63]. Anode is positively charged, and thus strongly attracts anions, which may block sites for water oxidation to produce hydroxyl free radicals. However, the anodic potential increased with increasing concentration CO_3_^2-^ (Fig 2C). It suggests that CO_3_^2-^ can be oxidized at lower anodic potential than water [45].

### The effect of pH

**Fig 3:**
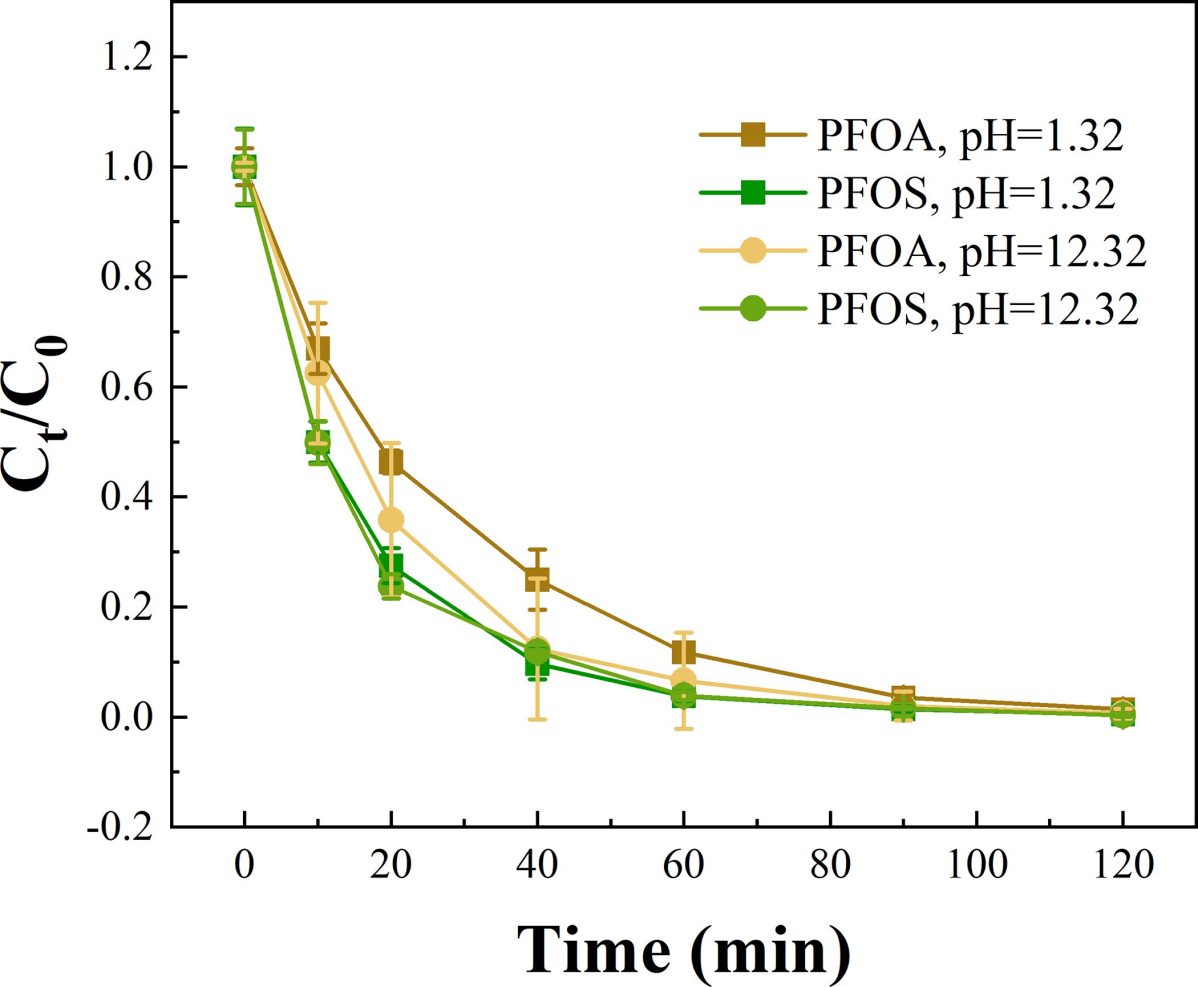
Concentration changes of PFOA/PFOS during EO on the Ti_4_O_7_ anodes at 10 mA·cm^-2^ in 100 mM H_3_PO_4_ (pH = 1.32) and 100 mM Na_3_PO_4_ (pH = 12.32).

An EO treatment experiment was conducted with 100 mM H_3_PO_4_ and 100 mM Na_3_PO_4_ as the supporting electrolyte solutions, which pH was 1.32 and 12.32, respectively. In The concentration change of PFOA/PFOS are shown in Fig 3. It is seen that PFOA degradation was inhibited when pH was low, while PFOS degradation was not affected by pH change from the acidic to the basic condition. The mass transfer rate of PFOA and PFOS was measured via limiting current method, with 4.20×10^−5^ m∙s^-1^ and 4.09×10^−5^ m∙s^-1^ obtained, respectively (S4 Text in S1 File), confirming that all the reactions studied in this work were kinetically controlled. In our previous studies, short-chain perfluoroalkyl acids (PFAAs) were observed as the intermediates of PFOA/PFOS degradation during EO tretament by titanium suboxide anodes and near complete F^-^ recovery was achieved [45, 64, 65]. The observed reaction rate constants *k*_*obs*,*PFAS*_ for PFOA/PFOS were calculated via fitting the pseudo-first order reaction model in all system, and the surface area normalized rate constant *k*_*SA*,*PFAS*_ can be calculated by *k*_*obs*,*PFAS*_ normalized to EESA (S5 Text in S1 File). The *k*_*SA*,*PFAS*_ for PFOA degradation was 2.96×10^−7^±4.24×10^−9^ m∙s^-1^ in 100 mM H_3_PO_4_ solution (pH = 1.32), about 22% lower than that in 100 mM Na_3_PO_4_ solution (pH = 12.32), which was 3.61×10^−7^±2.45×10^−8^ m∙s^-1^. It is likely that a larger fraction of the anionic form of PFOA because of deprotonation at higher pH facilitates mass transfer towards positively charged anode and oxidation. Furthermore, the *k*_*SA*,*PFAS*_ of PFOS degradation were 3.98×10^−7^±1.85×10^−8^ m∙s^-1^ in 100 mM H_3_PO_4_ solution (pH = 1.32), similar to that in 100 mM Na_3_PO_4_ solution (pH = 12.32), which is 4.01×10^−7^±3.79×10^−8^ m∙s^-1^. The pKa of PFOS is around -3.27 [66], and the dominant form is PFOS anion in the reaction solutions with pH at 1.32 or 12.32. Therefore, the degradation of PFOS was not affected at the pH values studied herein.

### The effect of nitrate ion

**Fig 4:**
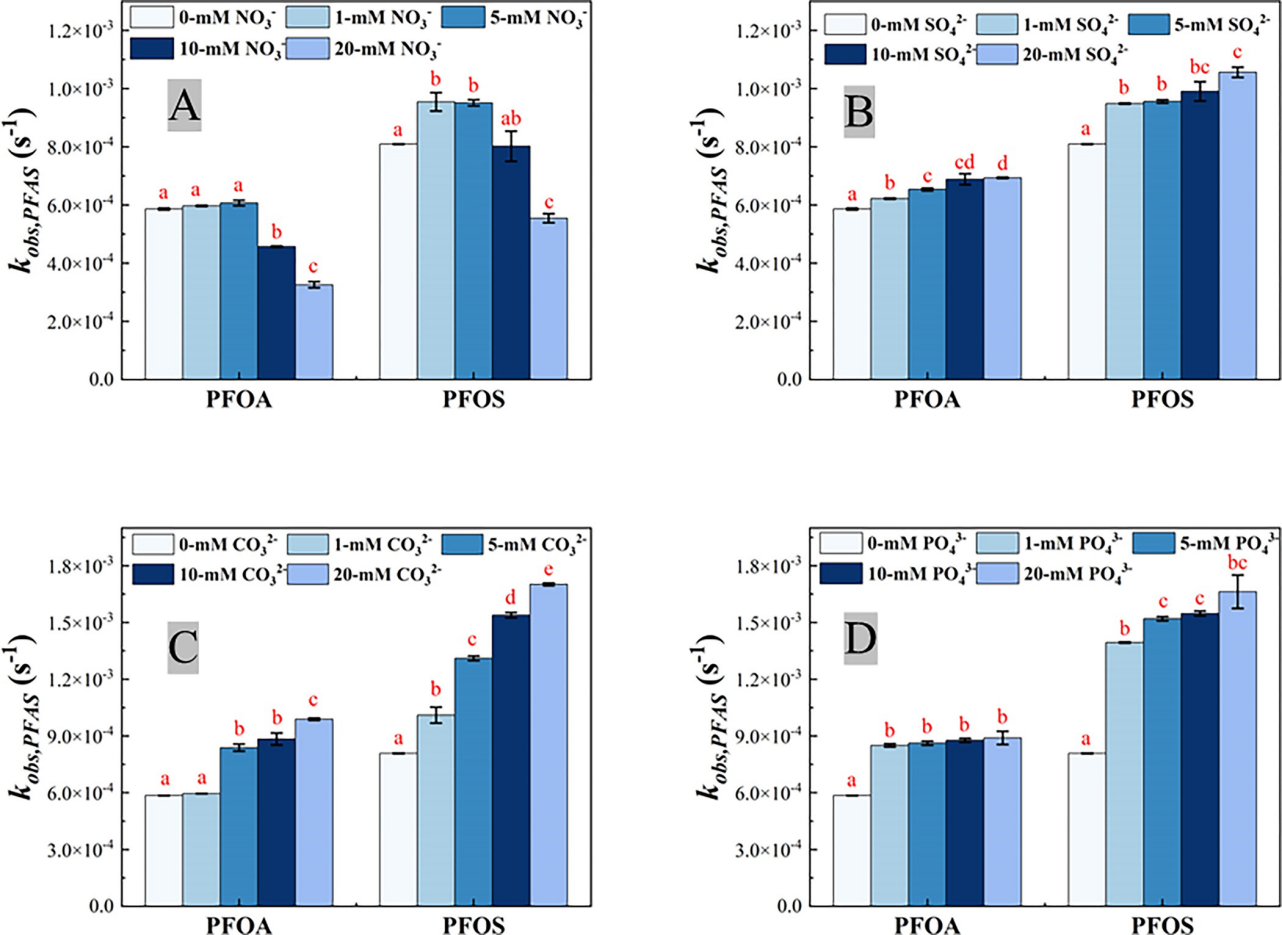
Observed reaction rate constant *k*_*obs*,*PFAS*_ for PFOA and PFOS degradation on the Ti_4_O_7_ anode in 100 mM NaClO_4_ with different concentrations of NO_3_^-^ (A), SO_4_2^-^ (B), CO_3_^2-^(C) and PO_4_^3-^ (D). The same letter indicates no statistical difference at α = 0.05, for the comparison of the *k*_*obs*,*PFAS*_ of PFOA/PFOS degradation of the same anions with different concentrations. Initial PFOA/PFOS concentration: 2 μM. Error bar represents standard deviations of replicates.

**Fig 5:**
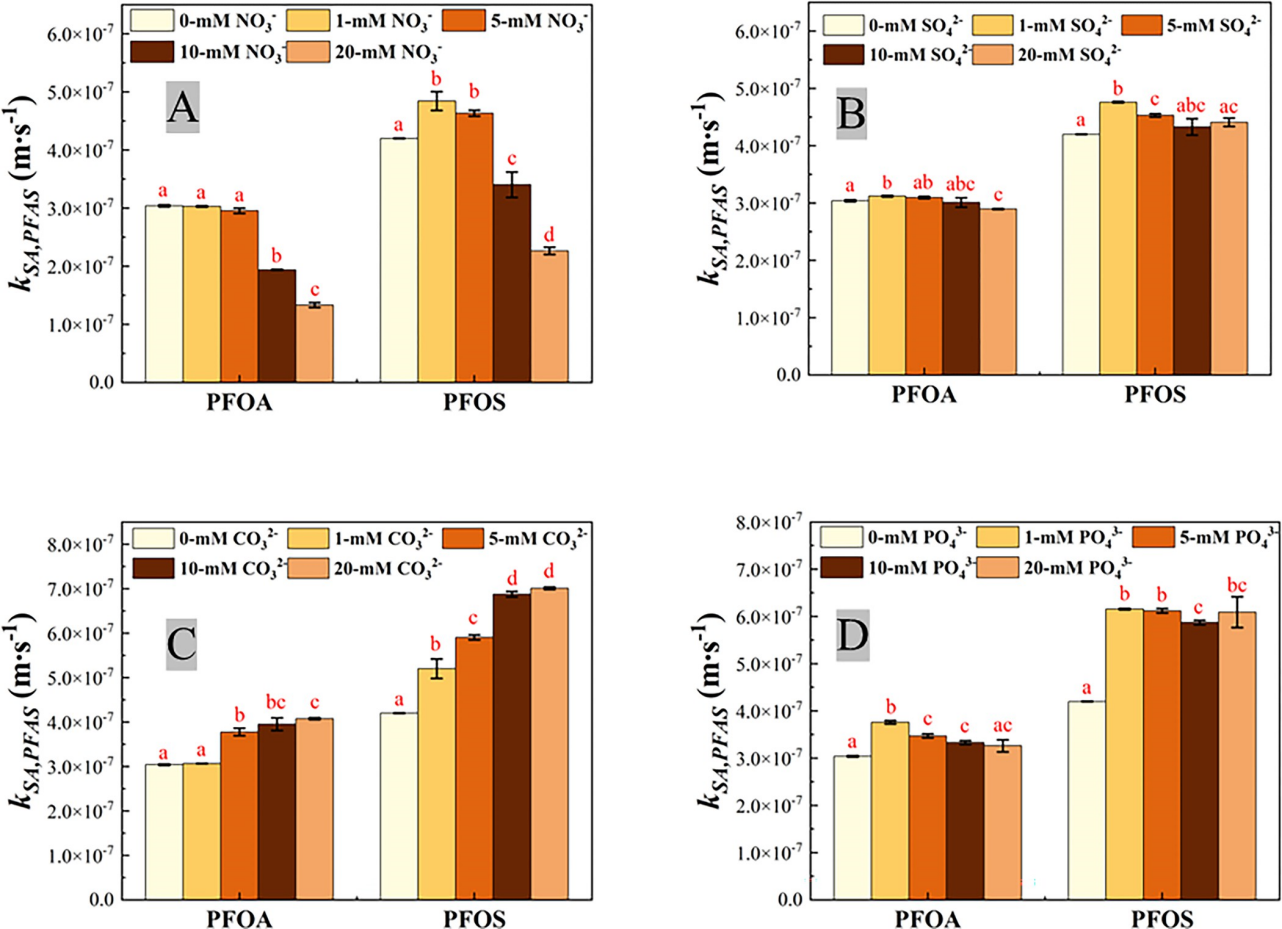
Surface area normalized reaction rate constant *k*_*SA*,*PFAS*_ for PFOA and PFOS degradation on the Ti_4_O_7_ anode in 100 mM NaClO_4_ with different concentrations of NO_3_^-^ (A), SO_4_2^-^ (B), CO_3_^2-^(C) and PO_4_^3-^ (D). The same letter indicates no statistical difference at α = 0.05, for the comparison of the *k*_*SAs*,*PFAS*_ of PFOA/PFOS degradation of the same anions with different concentrations. Initial PFOA/PFOS concentration: 2 μM. Error bar represents standard deviations of replicates.

The concentration profile of PFOA and PFOS during EO on the Ti_4_O_7_ anodes at current density 10 mA·cm^-2^ in 100 mM NaClO_4_ with varying concentrations of NaNO_3_ are presented in S2 Fig in S1 File. The addition of different anions, including NO_3_^-^, at different concentrations slightly increased the reaction solution conductivity (S3A Fig in S1 File), for example, the conductivity of 100 mM NaClO_4_ solution was 10.36 mS∙cm^-1^, and it rose to 12.07 mS∙cm^-1^ when 20 mM NO_3_^-^ was added into the system. However, the AP slightly decreased with different anions added at different concentrations (S3B Fig in S1 File). As shown in S2A Fig in S1 File, after 60 min EO treatment, 80.2% PFOA was removed from the reaction solution with 100 mM NaClO_4_ as the only electrolyte, while 83.4% and 86.6% PFOA was degraded when 5.0 mM and 1.0 mM NaNO_3_ was added, respectively, indicating promoted degradation of PFOA. However, such promotion effect was reversed with further increase of NO_3_^-^ concentration. The degradation of PFOA after 60 min was decreased to 69.0% and 61.2% when NO_3_^-^ concentration increased to 10 mM and 20 mM, respectively (S2A Fig in S1 File). PFOS degradation was faster than PFOA in the same reaction condition and exhibited a similar trend with PFOA. 93.7% PFOS was degraded on Ti_4_O_7_ anode after 60 mins without NO_3_^-^ added. The removal ratio increased to 98.1% and 97.8% when 1 mM and 5 mM NaNO_3_ were added to the reaction solution, respectively. However, when NO_3_^-^ concentration increased to 10 mM and 20 mM, the removal ratio decreased to 93.1% and 75.6%, respectively. The *k*_*obs*_ and *k*_*SA*_ values at varying NO_3_^-^ concentrations are reported in Figs 4 and 5, respectively, as well as SO_4_^2-^, PO_4_^3-^ and CO_3_^2-^, with results of ANOVA tests (S6 Text in S1 File) indicated. The *k*_*obs*_ and the *k*_*SA*_ for PFOA and PFOS in different electrolyte solutions are also summarized in S5 Table in S1 File.

As shown in Fig 4A, although the reaction rate constant (*k*_*obs*,*PFAS*_) did not vary much when 1 mM or 5 mM NO_3_^-^ added, there was a marked drop in *k*_*obs*,*PFAS*_ when NO_3_^-^ concentration increased to 10 mM and 20 mM. The *k*_*obs*,*PFAS*_ of PFOA decreasing from 6.07×10^−4^±9.43×10^−6^ s^-1^ to 3.26×10^−4^±1.06×10^−5^ s^-1^ when NO_3_^-^ concentration increased from 5 mM to 20 mM. Moreover, it is evident in Fig 5A that the surface area normalized rate constant *k*_*SA*,*PFAS*_ decreased along with increasing NO_3_^-^ in the background solution, indicating that PFOA/PFOS degradation was hindered in the presence of NO_3_^-^ with concentration higher than 10 mM. Such inhibition effect can be explained by the LSV results (Fig 2A). The anodic current decreased with increasing NO_3_^-^ concentration. It may suggest that NO_3_^-^ occupied the reactive sites on anode surface and hindered the electrolysis of water, which can be further confirmed by the quenching experiment using p-chlorobenzoic acid (pCBA) as the hydroxyl radical scavenger (see S7 Text in S1 File for detail). The steady-state hydroxyl radical concentration ([HO^∙^]_*ss*_) can be calculated via Equation S-7 in SI. Note that the EESA increased with increasing concentration of anions in the reaction solution (S1 Fig in S1 File). The [HO^∙^]_*ss*_ and EESA-normalized [HO^∙^]_*ss*_ are listed in S6 Table in S1 File. The EESA-normalized [HO^∙^]_*ss*_ was 1.20×10^−17^ M∙cm^-2^ in 100 mM NaClO_4_ solution, and it reduced to 1.19×10^−17^, 1.14×10^−17^, 6.66×10^−18^ and 6.02×10^−18^ M∙cm^-2^ when 1 mM, 5 mM, 10 mM and 20 mM NO_3_^-^ was added, respectively. Furthermore, nitrate ion can react with HO^∙^ to form nitrate radicals via reaction (1) that further consumeHO^∙^ in EO system, however, nitrate radicals do not have high reactivity to degrade PFOA and PFOS [67, 68]. Because HO^∙^ is essential to PFOA/PFOS degradation [41, 45], the presence of NO_3_^-^ led to inhibited degradation of PFOA/PFOS (Fig 5A) by reducing EESA-normalized steady-state concentration of HO^∙^ (S6 Table in S1 File).


$${HO}^{∙}+{NO}_{3}^{-}\to {HO}^{-}+{NO}_{3}^{∙}$$ (1)

### The effect of sulfate ion

The degradation curve of PFOA and PFOS on the Ti_4_O_7_ anode at 10 mA·cm^-2^ in 100 mM NaClO_4_ solution with varying concentrations of Na_2_SO_4_ are exhibited in S4 Fig in S1 File. PFOA/PFOS degradation increased slightly along with increasing concentration of SO_4_^2-^ added in the background. After 60 min EO treatment, 80.2% PFOA were removed with 100 mM NaClO_4_ as the only electrolyte, while 85.3%, 87.4%, 88.7% and 89.8% PFOA was degraded when 1.0 mM, 5.0 mM, 10 mM and 20 mM Na_2_SO_4_ was added, respectively (S4A Fig in S1 File). PFOS degradation showed a similar trend (S4B Fig in S1 File). The observed reaction rate constant *k*_*obs*,*PFAS*_ increased from 5.86×10^−4^±3.54×10^−6^ s^-1^ for PFOA and 8.09×10^−4^±1.18×10^−6^ s^-1^ for PFOS in the absence of SO_4_^2-^ to 6.93×10^−4^±2.36×10^−6^ s^-1^ for PFOA and 1.06×10^−3^±1.77×10^−5^ s^-1^ for PFOS in the presence of 20 mM SO_4_^2-^, respectively (Fig 4B, S5 Table in S1 File). However, the change of surface area normalized rate constant *k*_*SA*,*PFAS*_ of PFOA was nearly negligible, while the *k*_*SA*,*PFAS*_ of PFOS was slightly decreased when SO_4_^2-^ concentration was above 1mM, 3.04×10^−7^±1.83×10^−9^ m∙s^-1^ for PFOA and 4.20×10^−7^±6.12×10^−10^ m∙s^-1^ for PFOS in the absence of SO_4_^2-^ versus 2.89×10^−7^±9.84×10^−10^ m∙s^-1^ for PFOA and 4.41×10^−7^±7.38×10^−8^ m∙s^-1^ for PFOS in the presence of 20 mM SO_4_^2-^, respectively (Fig 5B, S5 Table in S1 File). The [HO^∙^]_*ss*_ was also quantified. The pCBA degradation slightly accelerated when higher concentration of SO_4_^2-^ was added (S5 Fig in S1 File), but EESA-normalized [HO^∙^]_*ss*_ remain at the same level (S7 Table in S1 File). It indicates that higher SO_4_^2-^ concentration promoted HO^∙^ formation, mainly because greater effective electroactive surface area (Table 1) became available for HO^∙^ formation, and thus facilitated PFOA/PFOS degradation.

### The effect of carbonate ion

S6 Fig in S1 File shows the concentration profile of PFOA/PFOS during 120 min EO treatment in 100-mM NaClO_4_ with different concentrations of Na_2_CO_3_. When current applied, the pH of reaction solution quickly increased and stabilized at 10.7–12.1 (S7 Fig in S1 File). Because the pKa_1_ and pKa2 of H_2_CO_3_ are 6.35 and 10.33, respectively [69], CO_3_^2-^ was the main form in the reaction solution rather than HCO_3_^-^. As shown in S6A Fig in S1 File, the PFOA/PFOS degradation was speeded up in the presence of CO_3_^2-^, with 81.1%, 94.6%, 95.2% and 96.8% PFOA removal achieved respectively with 1 mM, 5 mM, 10 mM and 20 mM CO_3_^2-^ in the reaction solution, compared to 80.2% PFOA removal in the absence of CO_3_^2-^ after 60 min EO treatment on the Ti_4_O_7_ anode. PFOS degradation exhibited a similar trend (S6B Fig in S1 File). Both *k*_*obs*,*PFAS*_ and *k*_*SA*,*PFAS*_ values are increased with increasing CO_3_^2-^ concentration in the background solution (Figs 4C and 5C), indicating that the presence of CO_3_^2-^ enhanced the degradation of PFOA/PFOS. The *k*_*obs*,*PFOA*_ were 5.96×10^−4^±1.18×10^−6^ s^-1^, 8.38×10^−4^±1.89×10^−5^ s^-1^, 8.84×10^−4^±3.18×10^−5^ s^-1^, 9.89×10^−4^±5.89×10^−6^ s^-1^ with 1 mM, 5 mM, 10 mM and 20 mM CO_3_^2-^ added, respectively, compared to 5.86×10^−4^±3.54×10^−6^ s^-1^ without Na_2_CO_3_ salt added. Likewise, the *k*_*SA*,*PFOA*_ increased from 3.04×10^−7^±1.83×10^−9^ m·s^-1^ in the absence Na_2_CO_3_ salt to 3.07×10^−7^±6.07×10^−10^ m·s^-1^, 3.77×10^−7^±8.49×10^−9^ m·s^-1^, 3.95×10^−7^±1.42×10^−8^ m·s^-1^, and 4.07×10^−7^±2.43×10^−9^ m·s^-1^ with 1 mM, 5 mM, 10 mM and 20 mM CO_3_^2-^ added, respectively. However, pCBA degradation cannot be detected when the concentration of CO_3_^2-^ was higher than 5 mM (S8 Fig in S1 File), indicating that CO_3_^2-^ can scavenge HO^∙^ generated in the EO system. CO_3_^2-^ can react with HO^∙^ to produce carbonate radical anion (${CO}_{3}^{∙-}$) by one-electron transfer between carbonate ion and hydroxyl free radical (HO^∙^) via reaction (2) [70]. CO_3_^2-^ can also be oxidized to ${CO}_{3}^{∙-}$ via losing one electron via reaction (3) [71], which can be evidenced by LSV result (Fig 2D). ${CO}_{3}^{∙-}$ is a strong oxidizing agent [72]. Compared to HO^∙^, ${CO}_{3}^{∙-}$ is more selective, targeting on the persistent organic pollutants, especially those that are electron-rich [73]. ${CO}_{3}^{∙-}$ was proved to have the capability of promoting the degradation of PFOA, especially in alkaline solution in both photochemical decomposition and sonochemical treatment [73–75]. If and how ${CO}_{3}^{∙-}$ may facilitates PFAS degradation during EO needs more exploration.


$${HO}^{∙}+{CO}_{3}^{2-}\to {HO}^{-}+{CO}_{3}^{∙-}(k=3.0\times 10^{8}\;M^{‐1}∙s^{‐1})$$ (2)

$${CO}_{3}^{2-}\to {CO}_{3}^{∙-}+{e_{aq}}^{-}$$ (3)

### The effect of phosphate ion

S9 Fig in S1 File shows the PFOA/PFOS concentration profiles during 120 min EO treatment in 100 mM NaClO_4_ with different concentrations of Na_3_PO_4_. Like CO_3_^2-^, increasing PO_4_^3-^ concentration promoted PFOA/PFOS degradation. After 60 min EO treatment, 80.2% PFOA was removed in 100 mM NaClO_4_ solution, while 94.6%, 94.8%, 96.0% and 96.9% PFOA was degraded when 1.0 mM, 5.0 mM, 10 mM and 20 mM Na_3_PO_4_ was added, respectively (S9A Fig in S1 File). PFOS degradation exhibited a similar trend (S9B Fig in S1 File). For PFOA degradation, the change of *k*_*obs*,*PFOA*_ was negligible with increasing Na_3_PO_4_ salt added, from 8.51×10^−4^±8.25×10^−6^ s^-1^ with 1 mM Na_3_PO_4_ added to 8.90×10^−4^±3.54×10^−5^ s^-1^ with 20 mM Na_3_PO_4_ added (Fig 4D), while *k*_*SA*,*PFOA*_ slightly decreased with increasing Na_3_PO_4_ salt added, from 3.76×10^−7^±3.64×10^−9^ m·s^-1^ with 1 mM Na_3_PO_4_ added to 3.26×10^−7^±1.29×10^−8^ s^-1^ with 20 mM Na_3_PO_4_ added (Fig 5D). However, for PFOS degradation, the *k*_*obs*,*PFOS*_ slightly increased with increasing Na_3_PO_4_ salt added, from 1.39×10^−3^±3.54×10^−6^ s^-1^ with 1 mM Na_3_PO_4_ added to 1.66×10^−3^±8.84×10^−5^ s^-1^ with 20 mM Na_3_PO_4_ added (Fig 4D), while the change of *k*_*SA*,*PFOS*_ was negligible with increasing Na_3_PO_4_ salt added, from 6.16×10^−7^±1.56×10^−9^ m·s^-1^ with 1 mM Na_3_PO_4_ added to 6.09×10^−7^±3.24×10^−8^ s^-1^ with 20 mM Na_3_PO_4_ added (Fig 5D). The pH change during EO treatment was shown in S10 Fig in S1 File. The pKa1, pKa2 and pKa3 of phosphoric acid are 2.15, 7.20 and 12.4, respectively [69]. When Na_3_PO_4_ concentrations increased from 1 mM to 20 mM, the pH increased from 12.04 to 12.46 at the end of 2 h experiment (S10 Fig in S1 File), and thus the dominant form changed from hydrogen phosphate ions (HPO_4_^2-^) to phosphate ions (PO_4_^3-^). HPO_4_^2-^ and PO_4_^3-^ can react with HO^∙^ to form hydrogen phosphate radicals and phosphate radicals via reaction (4) and (5), respectively. It is further evidenced by delayed pCBA degradation (S11 Fig in S1 File). Delayed pCBA degradation indicates that HO^∙^ was scavenged by HPO_4_^2-^ and PO_4_^3-^ in EO system [67, 76]. Phosphate radicals are selective oxidants whose reactivity are comparable with HO^∙^, which are capable of promoting organic compounds degradation via one-electron oxidation [67, 77, 78], as well as that of PFOA/PFOS during EO as shown in Fig 4D. Phosphate radicals may reacts preferentially with PFOS, leading to slight increase *k*_*obs*,*PFOS*_ (Fig 4D), but still needs further verification. However, when PO_4_^3-^ concentration increased further, excessive PO_4_^3-^ can couple with another PO_4_^3-^ to form peroxodiphosphate with two electrons extracted in alkaline solution via reaction (6) [79–81]. Peroxodiphosphate behaves as a soft oxidant in the oxidation of organics [82], which may not have the capability to attack PFOA/PFOS molecules. It is known that radical-mediated reactions play an important role in PFOA/PFOS degradation [41, 64]. Therefore, PFOA/PFOS degradation was accelerated (Fig 4D) due to larger EESA of Ti_4_O_7_ anode (Table 1) and strong oxidant formation of phosphate radicals when PO_4_^3-^ was added, but further increasing PO_4_^3-^ concentration led to more and more P_2_O_8_^4−^ formed on the anode surface, suppressing the formation of phosphate radicals, so that the EESA-normalized reaction rate constant of PFOA/PFOS tended to decrease with increasing PO_4_^3-^ (Fig 5D).


$${HPO}_{4}^{2-}+{HO}^{∙}\to {HPO}_{4}^{∙-}+{HO}^{-}$$ (4)

$${PO}_{4}^{3-}+{HO}^{∙}\to {PO}_{4}^{2-∙}+{HO}^{-}$$ (5)

$${{PO}_{4}}^{3-}+{{PO}_{4}}^{3-}\to P_{2}{O_{8}}^{4-}+2e^{-}$$ (6)

Overall, SO_4_^2-^, CO_3_^2-^ and PO_4_^3-^ promoted PFOA/PFOS degradation, while NO_3_^-^ hindered it, when its concentration was higher than 10 mM (Fig 4A). Such inhibition effect of NO_3_^-^ resulted from reduced EESA-normalized steady-state concentration of HO^∙^ (S6 Table in S1 File), which plays an vital role in PFOA/PFOS degradation [41]. The presence of SO_4_^2-^ accelerated PFOA/PFOS degradation due to higher HO^∙^ formation caused by enlarged EESA. CO_3_^2-^ reacted with HO^∙^ to form a strong and selective oxidant, ${CO}_{3}^{∙-}$, that has the capability of promoting PFOA/PFOS degradation by one-electron transfer reaction. Phosphate radicals, formed by the reaction between PO_4_^3-^/HPO_4_^2-^ and HO^∙^, was also able to promote PFOA/PFOS degradation, but peroxodiphosphate ion was generated and suppressed the formation of phosphate radicals, when PO_4_^3-^ was beyond 1 mM (Fig 5D), leading to delayed PFOA/PFOS degradation.

## Conclusions

The EO treatment of PFOA and PFOS, two representative PFASs, were conducted in reaction solutions with varying concentrations of different anions, including NO_3_^-^, SO_4_^2-^, CO_3_^2-^ and PO_4_^3-^. The acidity of the solution delayed PFOA degradation in relation to its acid dissociation behavior, but did not affect the PFOS degradation at the tested pH 1.32 and 12.32. NO_3_^-^ significantly hindered PFOA/PFOS degradation due to suppressed HO^∙^ generation, which plays an important role in PFAS degradation. PO_4_^3-^ slightly promoted PFOA/PFOS degradation, but phosphate radicals formation were suppressed by forming P_2_O_8_^4−^ simultaneously, delaying PFOA/PFOS degradation at high PO_4_^3-^ concentration. Increasing concentration of SO_4_^2-^ helped to increase EESA, and thus the observed degradation rates of PFOA/PFOS during EO, because more surface on the anode became accessible for EO reactions. The presence of CO_3_^2-^ greatly enhanced the degradation of PFOA/PFOS on account of the generation of ${CO}_{3}^{∙-}$, which can directly oxidize the perfluorinated carboxyl anions to form highly reactive radicals, and thus promoting the subsequent -CF_2_- unzipping processes. The information obtained in this study can help guide the implementation of Ti_4_O_7_-based EO treatment for PFAS removal in waters, particularly in industrial and municipal wastewater where large amounts of anions are present.

## Supporting information

S1 File (PDF)
