# Supplementary material for: The impact of anions on electrooxidation of perfluoroalkyl acids by porous Magnéli phase titanium suboxide anodes
Source: PLoS One. 2025 Jan 23;20(1):e0317696. doi: 10.1371/journal.pone.0317696 (PMC11756806; doi:10.1371/journal.pone.0317696)
Supplement: S1 File — (PDF) [file pone.0317696.s001.pdf]

Supporting Information

The impact of anions on electrooxidation of perfluoroalkyl acids by porous Magnéli phase titanium suboxide anodes

Yaye Wang[a], Yifei Wang[b], Shuping Dong[b], Qingguo Huang [b, *]

[a] Jiangsu Provincial Academy of Environmental Science, Nanjing 210036, People's Republic of China

[b] College of Agricultural and Environmental Sciences, Department of Crop and Soil Sciences, University of Georgia, Griffin, Georgia 30223, United States

[*] Corresponding author. E-mail addresses: qhuang@uga.edu.

19 pages

7 Texts

13 Figures

7 Tables

S1 Text. Detailed information of chemicals and reagents.

All chemicals used in the experiments were reagent grade and used as received. PFASs mixture standard solution (PFAC-24PAR and EPA-533PAR), isotopically labeled MPFAS mixture standard (MPFAS-24ES and EPA-533ES), Perfluoro-n-[$^{13}C_8$]octanoic acid (M8PFOA), Sodium perfluoro-[$^{13}C_8$]octanesulfonate (M8PFOS) were purchased from Wellington Laboratories (Ontario, Canada) and used as calibration standards and internal standard stock solution preparation. Perfluorooctanesulfonic acid (PFOS, 98%) was obtained from INDOFINE Chemical Company, Inc (Hillsborough, USA). Titanium suboxide (TiO_2), perfluorooctanoic acid (PFOA), sodium nitrate ($NaNO_3$), sodium carbonate (Na_2CO_3), phosphoric acid (H_3PO_4), p-Chlorobenzoic acid (pCBA, 99%) and sodium phosphate (Na_3PO_4) were obtained from Sigma Aldrich (St. Louis, USA). Sodium sulfate (Na_2SO_4) and ammonium acetate (CH_3COONH_4) were obtained from J.T.Baker Chemical (Phillipsburg, USA). Sodium perchlorate ($NaClO_4$), and HPLC-grade methanol were purchased from Fisher Scientific (Pittsburgh, USA). Milli-Q water (18.2 $M\Omega \cdot cm^{-1}$ resistivity, 25 ºC) prepared with Nanopure Barnstead purification system (Thermo Scientific, USA) was used in all experiments.

S2 Text. PFAS quantification.

PFAS quantification was performed on an ultra-performance liquid chromatography coupled with a triple-stage quadrupole mass spectrometer (ACQUITY UPLC-MS/MS, Xevo TQD, Waters Corp., USA). An Acquity UPLC BEH C18 column (2.10 mm × 50.0 mm, 1.70 μm) was used for UPLC separation with a mobile phase consisting of a water solution containing 2 mM ammonium acetate (A) and methanol containing 2 mM ammonium acetate (B). For optimizing resolution and peak shape, gradient UPLC method were used (Table S1). Electrospray ionization was operated in a negative mode for PFAS detection with the parameters set as capillary voltage at 2.70 kV, cone voltage 60 V, desolvation temperature at 350 ℃. Nitrogen (> 99.999% purity, Airgas) was used as the desolvation gas with the flow rates at 800 $L \cdot h^{-1}$. Multiple reaction monitoring (MRM) was used to detect all target compound. The target PFASs and their correspondent isotope-labeled internal standards are summarized in Table S3, and their transition and limit of detection are listed in Table S2. Quantification of PFAS concentration was achieved by the ratio between each PFAS and its correspondent isotope labeled standards in reference to a five-point calibration curve.

Linear calibrations must have an $r^2 \geq 0.99$ for each analyte, the peak areas of internal standards in water samples must be within 50-150% of the average area measured in the calibration standards.

S3 Text. Effective electroactive surface area measurement.

Total pore area does not necessarily reflect the actual pore area that is accessible for electrolyte during electrochemical process. Effective electroactive surface area (EESA) has been introduced to describe electroactive sites where EO reaction occurred, which can be calculated based on a method involving cyclic voltammetry (CV) of the porous Ti_4O_7 anodes in the different electrolyte solution at different scan rate. The scan rate-dependent voltametric charge q^* is a measure of electroactive sites. According to the literature, the total voltametric charge q_T^* is related to the total electroactive surface area, which can be divided in to two parts, q_O^* and q_I^*. q_O^* represent "outer" electroactive surface area that is exposed directly to electrolytes, while q_I^* described the "inner" surface which becomes less accessible to electrolyte when scan rate increases[1-3]. The ratio of EESA and total electroactive surface area equals to the ratio between q_O^* and q_T^*. These parameters can be extrapolated by suitable graphic correlation at scan rate approaching zero and infinite based on the CV results via the following equations.

$$q^* = q_O^* + k_1 v^{-1/2} \qquad \text{(S-1)}$$

$$(q^*)^{-1} = (q_T^*)^{-1} + k_2 v^{1/2} \qquad \text{(S-2)}$$

$$q_T^* = q_O^* + q_I^* \qquad \text{(S-3)}$$

where, v is the scan rate, k_1 and k_2 are constants. Roughness factor (RF) is the ratio of the total electroactive area per geometric surface area. It can be calculated by dividing the determined capacitance of the anode by the average double-layer capacitance of oxide electrodes (60 $\mu F\cdot cm^{-2}$). The q_T^*, q_O^*, q_I^*, RF and EESA of Ti_4O_7 anode calculated in different electrolyte solution are summarized in Table S4.

S4 Text. Mass transfer rate of PFOA/PFOS.

The limiting current technique was used to measure the mass transfer rate constant of PFOA and PFOS on Ti_4O_7 anode. 10 mM $K_4Fe(CN)_6$ and 10 mM $K_3Fe(CN)_6$ were dissolved in 100 mM KH_2PO_4 solution. A linear sweep potential with a slow scan rate (10 $mV\cdot s^{-1}$) was applied to the cell, and the corresponding current was measured. When the potential increased, the current was first increased along with the cell voltage, then remained stable when the current reached the

limiting current[4]. After hydrogen started to evolve, the current began to increase again (Figure S12). The mass transfer rate constant of $Fe(CN)_6^{4-}$ to different anode materials can be calculated by the following equation[4-6]:

$$k_{m,Fe(CN)_6^{4-}} = \frac{J_{lim}}{nFAC} \qquad \text{(S-4)}$$

Where J_{lim} is the limiting current density, $A \cdot m^{-2}$, n=1, F is the Faraday constant (96485 $C \cdot mol^{-1}$), C is the concentration of $Fe(CN)_6^{4-}$ in bulk solution, $mol \cdot m^{-3}$. Equation S-5 was used to calculate the mass transfer rate constant of PFOA and PFOS in different cells[6,7]:

$$k_{m,PFAS} = k_{m,Fe(CN)_6^{4-}} \times \left(\frac{D_{PFAS}}{D_{Fe(CN)_6^{4-}}}\right)^{\frac{2}{3}} \qquad \text{(S-5)}$$

D_{PFAS} and $D_{Fe(CN)_6^{4-}}$ are the diffusion coefficient of PFAS (4.90×10^{-10} $m^2 \cdot s^{-1}$ for PFOA, 4.70×10^{-10} $m^2 \cdot s^{-1}$ for PFOS)[8] and $Fe(CN)_6^{4-}$ (7.43×10^{-10} $m^2 \cdot s^{-1}$)[9]. Mass transfer rate of PFOA and PFOS were 4.20×10^{-5} $m \cdot s^{-1}$ and 4.09×10^{-5} $m \cdot s^{-1}$, respectively.

S5 Text. Calculation of observed reaction rate $k_{obs,PFAS}$ and surface area normalized rate constant $k_{SA,PFAS}$ for batch system.

The data of PFOA and PFOS concentration over time appeared to follow pseudo-first-order kinetic model. Therefore, the observed reaction rate constant $k_{obs,PFAS}$ of PFAS degradation was calculated by data fitting to equation (S-6):

$$-ln\frac{C_{t,PFAS}}{C_{0,PFAS}} = k_{obs,PFAS} \times t \qquad \text{(S-6)}$$

where $C_{0,PFAS}$ is the substrate concentration at time zero ($mol \cdot L^{-1}$); $C_{t,PFAS}$ is the substrate concentration ($mol \cdot L^{-1}$) at time t (s), PFAS in the subscript can be PFOA or PFOS. The observed reaction rete constant was obtained from the slope of the linear relationship between time t and the negative natural logarithm of $\frac{C_{t,PFAS}}{C_{0,PFAS}}$ at time t, while the standard deviation was calculated from the rate constants obtained by each of the two replicate data sets. This is illustrated in Figure S13 using PFOA degradation in 100 mM H_3PO_4 as an example, with the rate constants obtained from each of the two replicate data sets being 6.09×10^{-4} s^{-1} and 5.96×10^{-4} s^{-1}, while $k_{obs,PFOA}$ of

6.02×10^{-4} s^{-1} was calculated from the average of the replicates, with the standard deviation of 8.63×10^{-6} s^{-1}.

It should be noted that different anodes had different effective electroactive surface area to solution volume ratio. Therefore, PFAS degradation rate constant normalized to effective electroactive surface area ($k_{SA,PFAS}$) was calculated to facilitate the comparison of reactivity in different electrolyte solutions. The surface area normalized reaction rate constant ($k_{SA,PFAS}$) was calculated by the following equation (S-7)[10]:

$$k_{SA,PFAS} = k_{obs,PFAS} \times \frac{V}{S} \qquad \text{(S-7)}$$

Where S is the effective electroactive surface area of the anode (m^2), V is the volume of the reaction solution (m^3) corresponding to the effective electroactive surface area, PFAS in the subscript can be PFOA or PFOS.

S6 Text. Statistical analysis.

One-way ANOVA was performed on $k_{obs,PFAS}$ and $k_{SA,PFAS}$ of PFOA/PFOS degradation in solutions of the same anions added at different concentrations, followed by a least significant difference test (α = 0.05). The results are indicated in Figure 4 and 5 for $k_{obs,PFAS}$ and $k_{SA,PFAS}$. Respectively.

S7 Text. Steady-state hydroxyl radical concentration ($[\mathrm{HO}^{\cdot}]_{ss}$).

Steady-state hydroxyl radical concentration ($[\mathrm{HO}^{\cdot}]_{ss}$) was probed by p-chlorobenzoic acid (pCBA), as it reacts rapidly with hydroxyl radical (HO$^{\cdot}$) generated on the anode surface via (R-1) and the direct electron transfer (DET) process of pCBA on Ti_4O_7 anode is not significant that appropriate for $[\mathrm{HO}^{\cdot}]_{ss}$ quantification[11].

$$\mathrm{HO}^{\cdot} + \mathrm{pCBA} \rightarrow \mathrm{intermediates} \qquad \text{(R-1)}$$

$$-\frac{d[\mathrm{pCBA}]}{dt} = k_{obs,\mathrm{pCBA}}[\mathrm{pCBA}] = k_{\mathrm{HO}^{\cdot},\mathrm{pCBA}}[\mathrm{pCBA}][\mathrm{HO}^{\cdot}]_{ss} \qquad \text{(S-8)}$$

$$[\mathrm{HO}^{\cdot}]_{ss} = \frac{k_{obs,\mathrm{pCBA}}}{k_{\mathrm{HO}^{\cdot},\mathrm{pCBA}}} \qquad \text{(S-9)}$$

The second-order rate constant for ·OH reacting with pCBA ($k_{\mathrm{HO^{\cdot}},\mathrm{pCBA}}$) in (S-8) is $5.2 \times 10^{9}\ M^{-1} \cdot s^{-1}$ [12]. The pseudo first-order rate constant of pCBA ($k_{obs,\mathrm{pCBA}}$) was measuring by pCBA degradation experiment with 10 μM pCBA in 100-mM Na_2SO_4 and different concentrations of different anoins as supporting electrolyte.

S1 Table. UPLC Method.

Time (min)	Flow rate ($mL\cdot min^{-1}$)	% A	% B
**Initial**	0.3	95	5
**5.00**	0.3	95	5
**1.50**	0.3	60	40
**10.00**	0.3	20	80
**11.00**	0.3	5	95
**14.00**	0.3	95	5
**16.00**	0.3	95	5

S2 Table. MS transition and limit of detection for all target PFASs analyzed in this study.

Compound Name	Parent (m/z)	Daughter (m/z)	Detection limit (ppb)
**PFOA**	412.86	369.00	0.51
**PFOS**	498.78	80.20	0.50

S3 Table. The target PFASs and correspondent isotope-labeled internal standards.

Target	Acronym	Isotope-labeled internal standards	Acronym
Perfluorooctanoic acid	PFOA	Perfluoro-n-[$^{13}C_8$]octanoic acid	$^{13}C_8$-PFOA
Perfluorooctanesulfonic acid	PFOS	Perfluoro-[$^{13}C_8$]octanesulfonate	$^{13}C_8$-PFOS

S4 Table. Total, outer and inner charge values and EESA of Ti_4O_7 anodes in different electrolyte solutions.

Electrolyte	q_T^* ($mC\cdot cm^{-2}$)	q_O^* ($mC\cdot cm^{-2}$)	q_I^* ($mC\cdot cm^{-2}$)	RF	EESA (cm^2)
**100 mM H_3PO_4**	156.25	36.70	119.55	535.00	2035.71
**100 mM Na_3PO_4**	149.25	33.78	115.48	550.00	2016 .33
**100 mM $NaClO_4$**	101.01	29.43	71.59	408.33	1927.00
**100 mM $NaClO_4$+1 mM $NaNO_3$**	80.00	26.66	53.34	365.00	1970.66
**100 mM $NaClO_4$+5 mM $NaNO_3$**	78.74	27.10	51.64	368.33	2053.36
**100 mM $NaClO_4$+10 mM $NaNO_3$**	85.47	30.39	55.74	411.67	2356.55
**100 mM $NaClO_4$+20 mM $NaNO_3$**	86.21	30.46	55.09	425.00	2447.65

**100 mM $NaClO_4$+1 mM Na_2SO_4**	97.09	29.14	67.95	410.00	1993.34
**100 mM $NaClO_4$+5 mM Na_2SO_4**	96.15	30.07	66.09	416.67	2110.56
**100 mM $NaClO_4$+10 mM Na_2SO_4**	93.46	31.18	62.28	423.33	2287.86
**100 mM $NaClO_4$+20 mM Na_2SO_4**	90.91	32.01	58.90	420.00	2395.38
**100 mM $NaClO_4$+1 mM Na_3PO_4**	103.09	31.79	71.30	453.33	2264.62
**100 mM $NaClO_4$+5 mM Na_3PO_4**	96.15	33.00	63.50	451.67	2484.64
**100 mM $NaClO_4$+10 mM Na_3PO_4**	90.91	32.65	57.91	448.33	2636.31
**100 mM $NaClO_4$+20 mM Na_3PO_4**	89.29	33.69	55.60	446.67	2730.26
**100 mM $NaClO_4$+1 mM Na_2CO_3**	103.09	29.21	73.89	423.33	1942.86
**100 mM $NaClO_4$+5 mM Na_2CO_3**	93.46	30.38	63.08	418.33	2221.25
**100 mM $NaClO_4$+10 mM Na_2CO_3**	90.91	30.63	60.28	410.00	2238.04
**100 mM $NaClO_4$+20 mM Na_2CO_3**	86.21	31.13	55.08	415.00	2427.57

S5 Table. The $k_{obs,PFAS}$ and $k_{SA,PFAS}$ corresponding to the Ti_4O_7 anodes in different electrolyte solutions.

Electrolyte		$k_{obs,PFOA}$ (s^{-1})	CLD[i]	$k_{obs,PFOS}$ (s^{-1})	CLD	$k_{SA,PFOA}$ ($m \cdot s^{-1}$)	CLD	$k_{SA,PFOS}$ ($m \cdot s^{-1}$)	CLD
**100 mM $NaClO_4$**		$5.86 \times 10^{-4} \pm 3.54 \times 10^{-6}$	a	$8.09 \times 10^{-4} \pm 1.18 \times 10^{-6}$	a	$3.04 \times 10^{-7} \pm 1.83 \times 10^{-9}$	a	$4.20 \times 10^{-7} \pm 6.12 \times 10^{-10}$	a
**Add $NaNO_3$**	1 mM	$5.97 \times 10^{-4} \pm 2.36 \times 10^{-6}$	a	$9.54 \times 10^{-4} \pm 3.18 \times 10^{-5}$	b	$3.03 \times 10^{-7} \pm 1.20 \times 10^{-9}$	a	$4.84 \times 10^{-7} \pm 1.61 \times 10^{-8}$	b
	5 mM	$6.07 \times 10^{-4} \pm 9.43 \times 10^{-6}$	a	$9.51 \times 10^{-4} \pm 1.06 \times 10^{-5}$	b	$2.95 \times 10^{-7} \pm 4.59 \times 10^{-9}$	a	$4.63 \times 10^{-7} \pm 5.17 \times 10^{-9}$	b
	10 mM	$4.57 \times 10^{-4} \pm 2.36 \times 10^{-6}$	b	$8.02 \times 10^{-4} \pm 5.19 \times 10^{-5}$	ab	$1.94 \times 10^{-7} \pm 1.00 \times 10^{-9}$	b	$3.40 \times 10^{-7} \pm 2.20 \times 10^{-8}$	c
	20 mM	$3.26 \times 10^{-4} \pm 1.06 \times 10^{-5}$	c	$5.54 \times 10^{-4} \pm 1.53 \times 10^{-5}$	c	$1.33 \times 10^{-7} \pm 4.33 \times 10^{-9}$	c	$2.26 \times 10^{-7} \pm 6.26 \times 10^{-9}$	d
**Add Na_2SO_4**	1 mM	$6.22 \times 10^{-4} \pm 2.36 \times 10^{-6}$	b	$9.48 \times 10^{-4} \pm 2.36 \times 10^{-6}$	b	$3.12 \times 10^{-7} \pm 1.18 \times 10^{-9}$	b	$4.80 \times 10^{-7} \pm 2.96 \times 10^{-9}$	b
	5 mM	$6.53 \times 10^{-4} \pm 4.71 \times 10^{-6}$	c	$9.56 \times 10^{-4} \pm 5.89 \times 10^{-6}$	b	$3.10 \times 10^{-7} \pm 2.23 \times 10^{-9}$	ab	$4.53 \times 10^{-7} \pm 2.79 \times 10^{-9}$	c
	10 mM	$6.88 \times 10^{-4} \pm 1.89 \times 10^{-5}$	cd	$9.90 \times 10^{-4} \pm 3.30 \times 10^{-5}$	bc	$3.01 \times 10^{-7} \pm 8.24 \times 10^{-9}$	abc	$4.33 \times 10^{-7} \pm 1.44 \times 10^{-8}$	abc
	20 mM	$6.93 \times 10^{-4} \pm 2.36 \times 10^{-6}$	d	$1.06 \times 10^{-3} \pm 1.77 \times 10^{-5}$	c	$2.89 \times 10^{-7} \pm 9.84 \times 10^{-10}$	c	$4.41 \times 10^{-7} \pm 7.38 \times 10^{-8}$	ac
**Add Na_2CO_3**	1 mM	$5.96 \times 10^{-4} \pm 1.18 \times 10^{-6}$	a	$1.01 \times 10^{-3} \pm 4.24 \times 10^{-5}$	b	$3.07 \times 10^{-7} \pm 6.07 \times 10^{-10}$	a	$5.20 \times 10^{-7} \pm 2.18 \times 10^{-8}$	b
	5 mM	$8.38 \times 10^{-4} \pm 1.89 \times 10^{-5}$	b	$1.31 \times 10^{-3} \pm 1.30 \times 10^{-5}$	c	$3.77 \times 10^{-7} \pm 8.49 \times 10^{-9}$	b	$5.90 \times 10^{-7} \pm 5.84 \times 10^{-8}$	c
	10 mM	$8.84 \times 10^{-4} \pm 3.18 \times 10^{-5}$	b	$1.54 \times 10^{-3} \pm 1.41 \times 10^{-5}$	d	$3.95 \times 10^{-7} \pm 1.42 \times 10^{-8}$	bc	$6.87 \times 10^{-7} \pm 6.32 \times 10^{-9}$	d
	20 mM	$9.89 \times 10^{-4} \pm 5.89 \times 10^{-6}$	c	$1.70 \times 10^{-3} \pm 7.07 \times 10^{-6}$	e	$4.07 \times 10^{-7} \pm 2.43 \times 10^{-9}$	c	$7.01 \times 10^{-7} \pm 2.91 \times 10^{-9}$	d
**Add Na_3PO_4**	1 mM	$8.51 \times 10^{-4} \pm 8.25 \times 10^{-6}$	b	$1.39 \times 10^{-3} \pm 3.54 \times 10^{-6}$	b	$3.76 \times 10^{-7} \pm 3.64 \times 10^{-9}$	b	$6.16 \times 10^{-7} \pm 1.56 \times 10^{-9}$	b
	5 mM	$8.63 \times 10^{-4} \pm 1.06 \times 10^{-5}$	b	$1.52 \times 10^{-3} \pm 1.18 \times 10^{-5}$	c	$3.47 \times 10^{-7} \pm 4.27 \times 10^{-8}$	c	$6.12 \times 10^{-7} \pm 4.74 \times 10^{-9}$	b

10 mM	$8.78 \times 10^{-4} \pm 1.06 \times 10^{-5}$	b	$1.55 \times 10^{-3} \pm 1.30 \times 10^{-5}$	c	$3.33 \times 10^{-7} \pm 4.02 \times 10^{-9}$	c	$5.87 \times 10^{-7} \pm 4.92 \times 10^{-9}$	c
20 mM	$8.90 \times 10^{-4} \pm 3.54 \times 10^{-5}$	b	$1.66 \times 10^{-3} \pm 8.84 \times 10^{-5}$	bc	$3.26 \times 10^{-7} \pm 1.29 \times 10^{-8}$	ac	$6.09 \times 10^{-7} \pm 3.24 \times 10^{-8}$	bc

[i]: Compact letter display, the same letter indicates no statistical difference at α=0.05.

S6 Table. Steady-state hydroxyl radical concentration $[\mathrm{HO}^{\cdot}]_{ss}$ and EESA-normalized $[\mathrm{HO}^{\cdot}]_{ss}$ on Ti_4O_7 anode in 100-mM $NaClO_4$ with different concentrations of NO_3^-.

Anode	$[\mathrm{HO}^{\cdot}]_{ss}$ (M)	EESA-normalized $[\mathrm{HO}^{\cdot}]_{ss}$ (M·cm^{-2})
**0 mM**	2.32×10^{-14}	1.20×10^{-17}
**1 mM**	2.35×10^{-14}	1.19×10^{-17}
**5 mM**	2.34×10^{-14}	1.14×10^{-17}
**10 mM**	1.57×10^{-14}	6.66×10^{-18}
**20 mM**	1.47×10^{-14}	6.02×10^{-18}

S7 Table. Steady-state hydroxyl radical concentration $[\mathrm{HO}^{\cdot}]_{ss}$ and EESA-normalized $[\mathrm{HO}^{\cdot}]_{ss}$ on Ti_4O_7 anode in 100-mM $NaClO_4$ with different concentrations of SO_4^{2-}.

Anode	$[\mathrm{HO}^{\cdot}]_{ss}$ (M)	EESA-normalized $[\mathrm{HO}^{\cdot}]_{ss}$ (M·cm^{-2})
**0 mM**	2.32×10^{-14}	1.20×10^{-17}
**1 mM**	2.35×10^{-14}	1.18×10^{-17}
**5 mM**	2.48×10^{-14}	1.18×10^{-17}
**10 mM**	2.68×10^{-14}	1.17×10^{-17}
**20 mM**	2.78×10^{-14}	1.16×10^{-17}

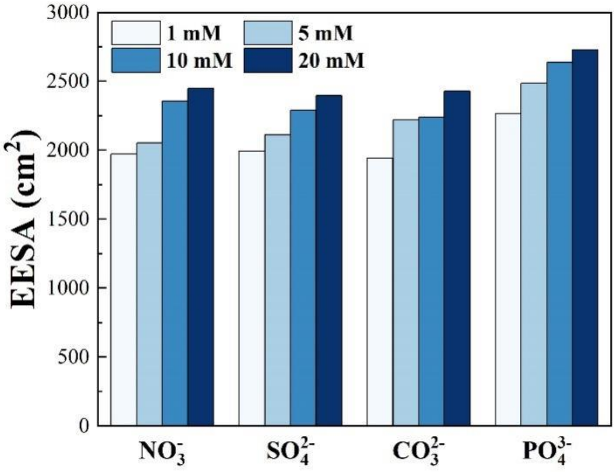

S1 Fig. The EESA of Ti_4O_7 anode in 100 mM $NaClO_4$ with different anions added.

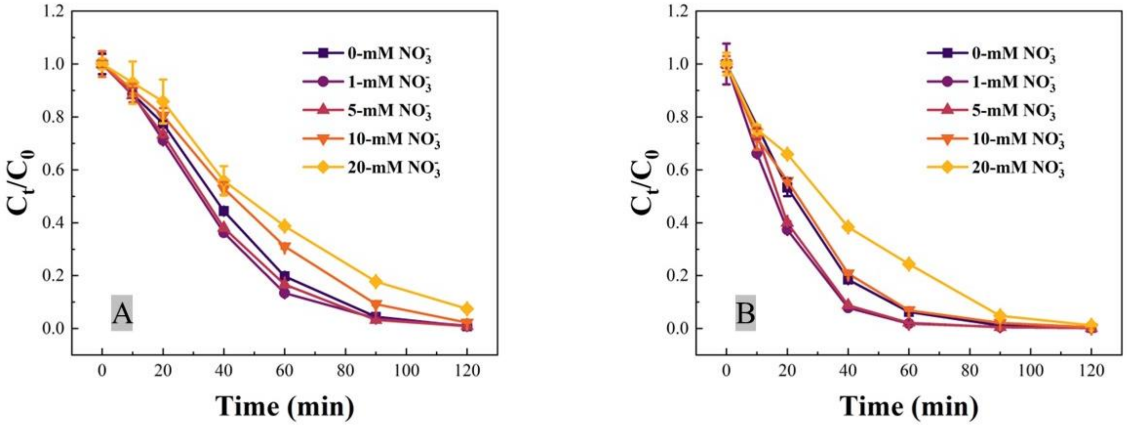

S2 Fig. The concentration profile of PFOA (A) and PFOS (B) during EO treatment at 10 mA·cm^{-2} on the Ti_4O_7 anode in 100 mM $NaClO_4$ with varying concentration of $NaNO_3$. Initial PFOA/PFOS concentration: 2.0 μM. Error bar represents standard deviations of replicates.

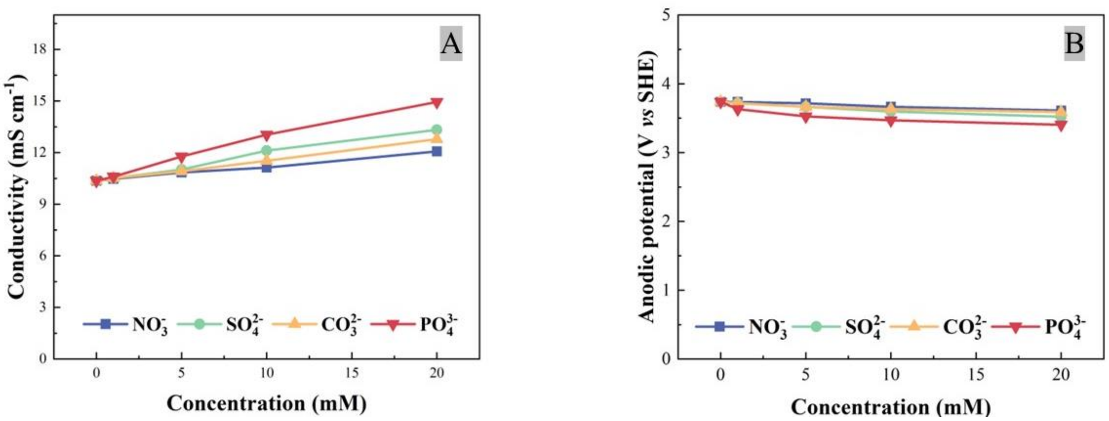

S3 Fig. The conductivity of 100 mM $NaClO_4$ with different sodium salts spiked (A) and anodic potential on the Ti_4O_7 anode in 100 mM $NaClO_4$ with different sodium salts spiked (B). Initial PFOA/PFOS concentration: 2.0 μM, current density = 10 mA·cm^{-2}.

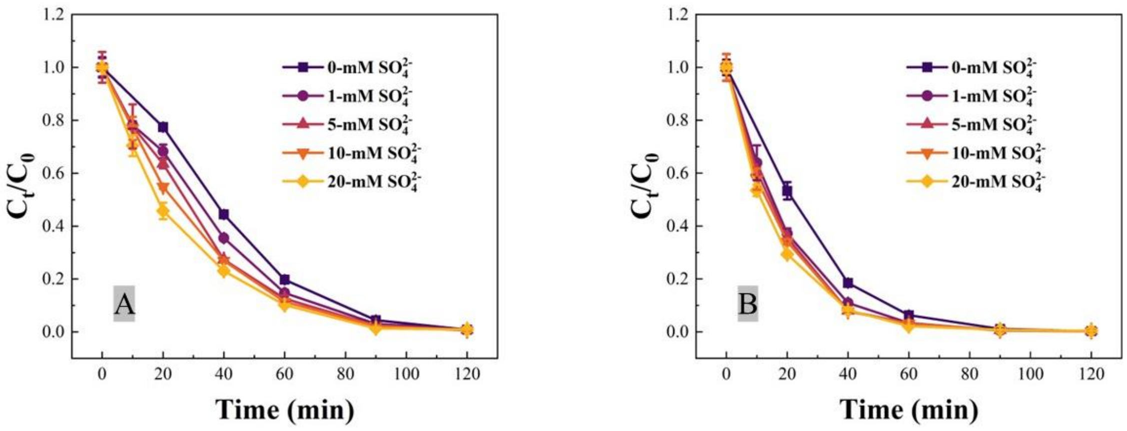

S4 Fig. The concentration profile of PFOA (A) and PFOS (B) during EO treatment at 10 mA·cm^{-2} on the Ti_4O_7 anode in 100 mM $NaClO_4$ with varying concentration of Na_2SO_4. Initial PFOA/PFOS concentration: 2.0 μM. Error bar represents standard deviations of replicates.

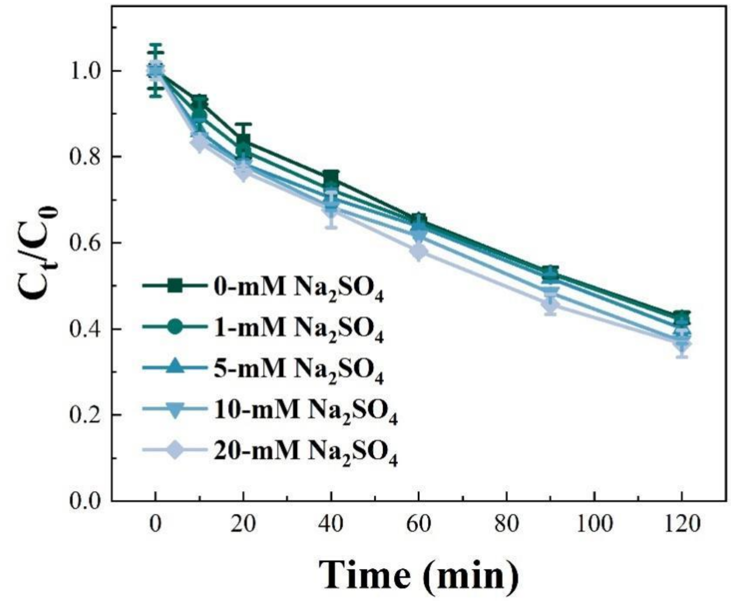

S5 Fig. pCBA degradation on Ti_4O_7 anode in 100 mM $NaClO_4$ with different concentrations of Na_2SO_4. Initial pCBA concentration: 10 μM, current density = 10 mA·cm^{-2}. Error bar represents standard deviations of replicates

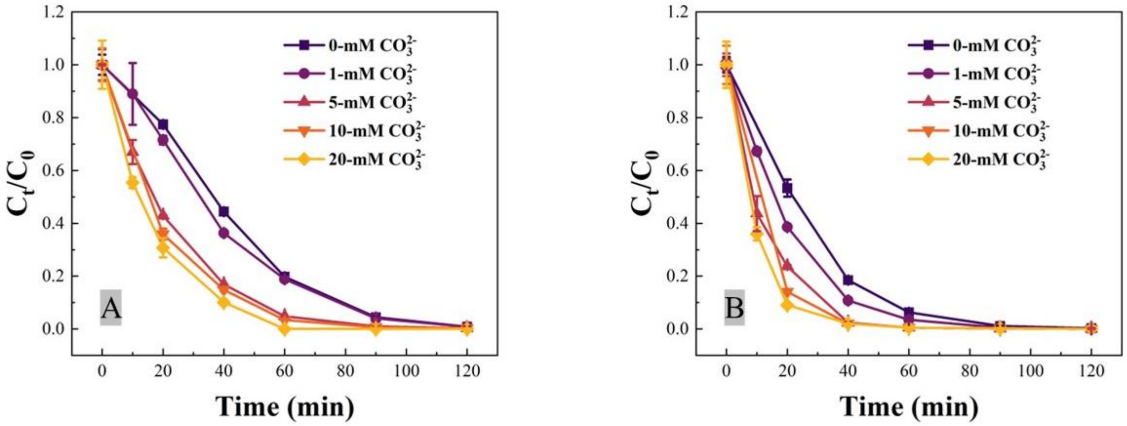

S6 Fig. The concentration profile of PFOA (A) and PFOS (B) during EO treatment at 10 mA·cm^{-2} on the Ti_4O_7 anode in 100 mM $NaClO_4$ with varying concentration of Na_2CO_3. Initial PFOA/PFOS concentration: 2.0 μM. Error bar represents standard deviations of replicates.

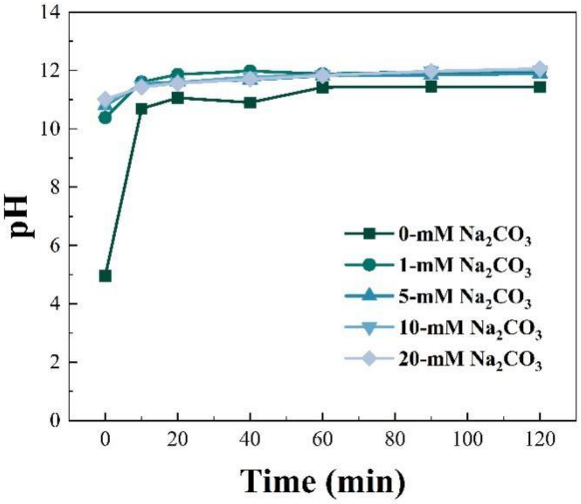

S7 Fig. The pH change during EO treatment on the Ti_4O_7 anode in 100 mM $NaClO_4$ with different concentration of Na_2CO_3. Initial PFOA/PFOS concentration: 2.0 µM, current density = 10 $mA \cdot cm^{-2}$.

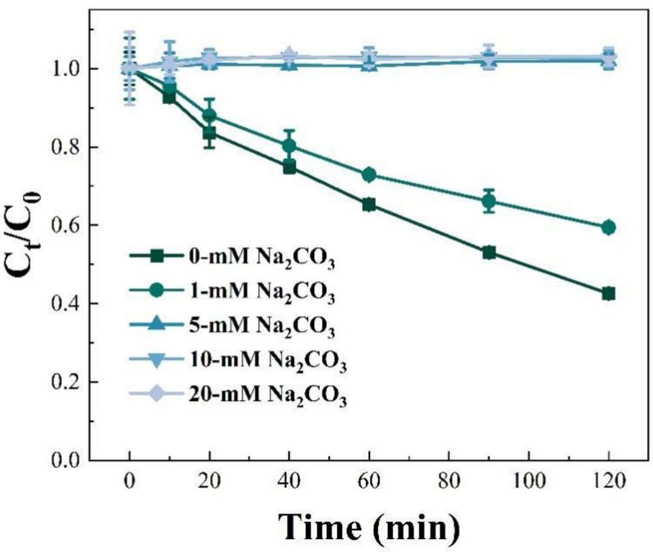

S8 Fig. pCBA degradation on Ti_4O_7 anode in 100 mM $NaClO_4$ with different concentrations of Na_2CO_3. Initial pCBA concentration: 10 µM, current density = 10 $mA \cdot cm^{-2}$. Error bar represents standard deviations of replicates.

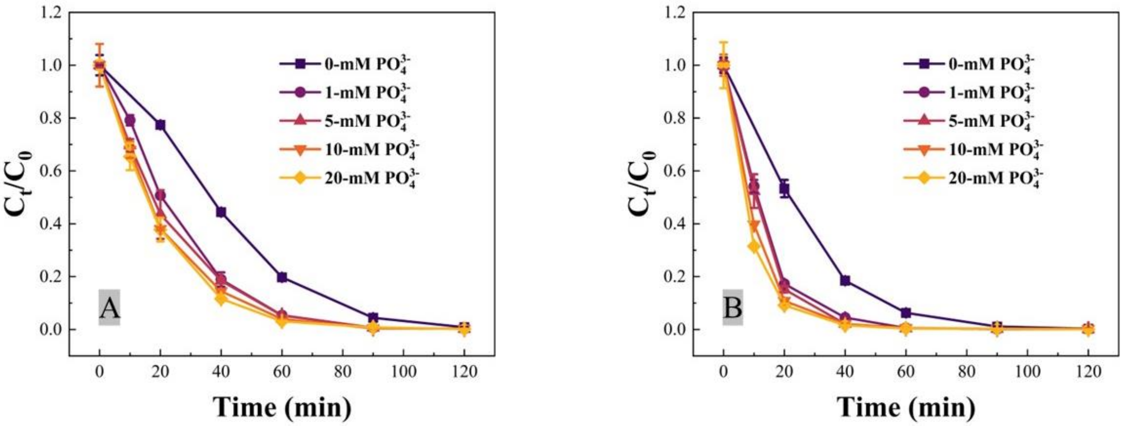

S9 Fig. The concentration profile of PFOA (A) and PFOS (B) during EO treatment at 10 mA·cm^{-2} on the Ti_4O_7 anode in 100 mM $NaClO_4$ with varying concentration of Na_3PO_4. Initial PFOA/PFOS concentration: 2.0 μM. Error bar represents standard deviations of replicates.

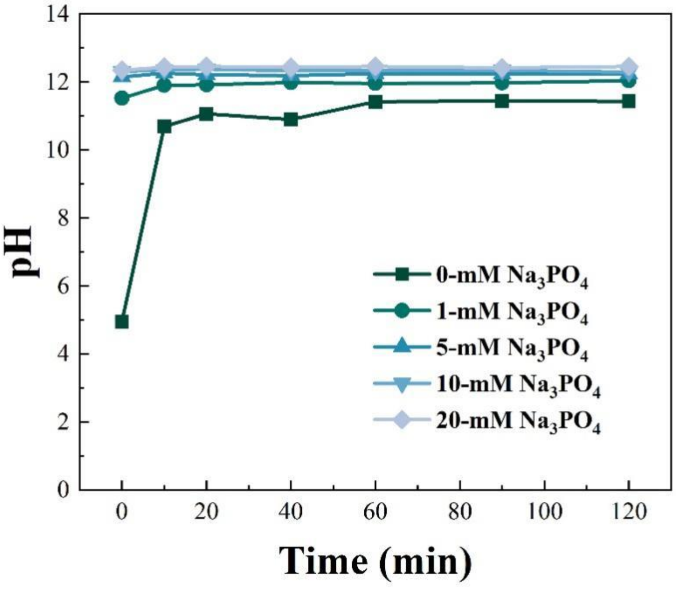

S10 Fig. The pH change during EO treatment on the Ti_4O_7 anode in 100 mM $NaClO_4$ with different concentration of Na_3PO_4. Initial PFOA/PFOS concentration: 2.0 μM, current density = 10 mA·cm^{-2}.

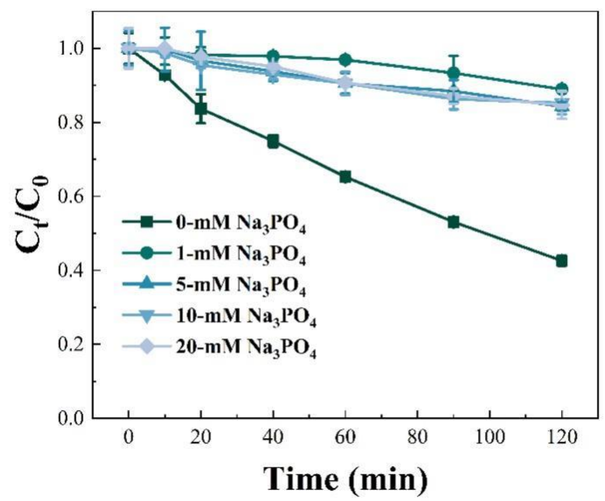

S11 Fig. pCBA degradation on Ti_4O_7 anode in 100 mM $NaClO_4$ with different concentrations of Na_3PO_4. Initial pCBA concentration: 10 μM, current density = 10 mA·cm^{-2}. Error bar represents standard deviations of replicates.

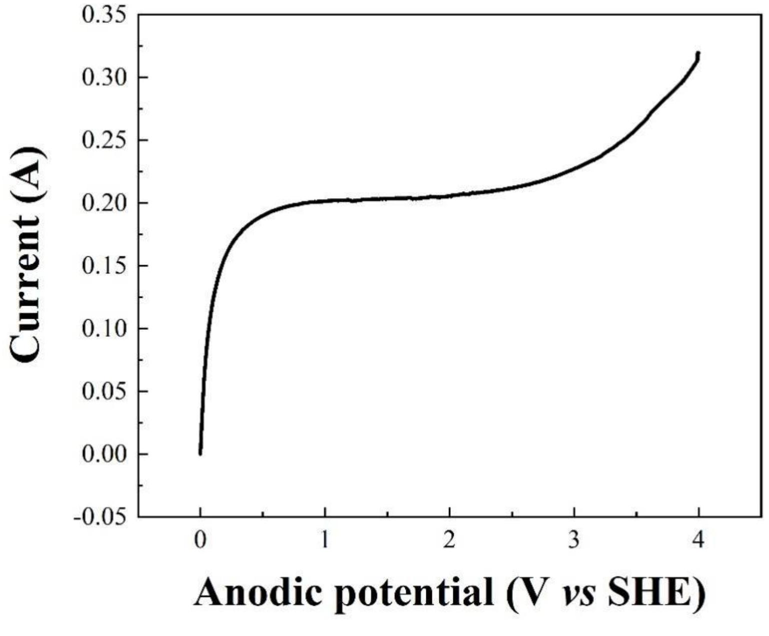

S12 Fig. Linear sweep voltammogram of 10 mM $K_4Fe(CN)_6$ on Ti_4O_7 anode in 100 mM KH_2PO_4 solution.

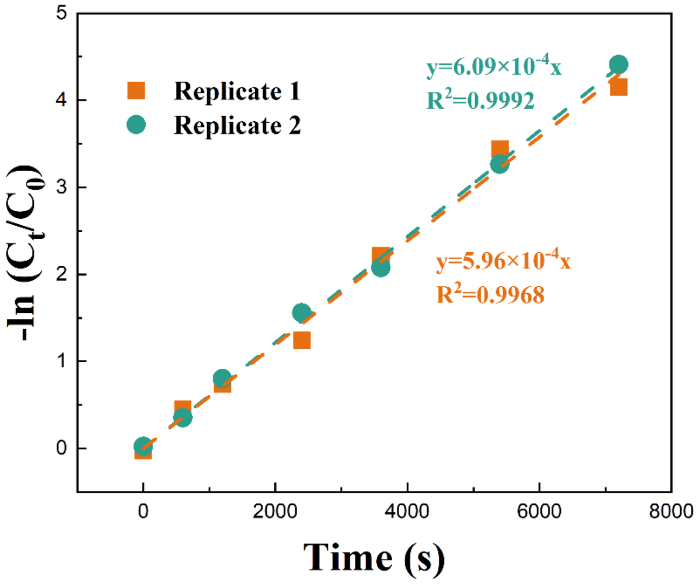

S13 Fig. The correlation between time t and negative natural logarithm of the $\frac{C_{t,PFOA}}{C_{0,PFOA}}$. Initial PFOA concentration: 2.0 μM, current density = 10 mA·cm^{-2}.

Reference

1 Ardizzone, S., Fregonara, G. & Trasatti, S. "Inner" and "outer" active surface of RuO2 electrodes. *Electrochimica Acta* **35**, 263-267, doi:https://doi.org/10.1016/0013-4686(90)85068-X (1990).

2 Xu, L. & Scantlebury, J. Electrochemical Surface Characterization of IrO2-Ta2O5 Coated Titanium Electrodes in Na2SO4 Solution. *Journal of The Electrochemical Society* **150**, B288-B293, doi:10.1149/1.1574033 (2003).

3 Zhao, W., Xing, J., Chen, D., Bai, Z. & Xia, Y. Study on the performance of an improved Ti/SnO2-Sb2O3/PbO2 based on porous titanium substrate compared with planar titanium substrate. *RSC Advances* **5**, 26530-26539, doi:10.1039/C4RA13492C (2015).

4 Scott, K. & Lobato, J. Determination of a Mass-Transfer Coefficient Using the Limiting-Current Technique. *The Chemical Educator* **7**, 214-219, doi:10.1007/s00897020579a (2002).

5 Cañizares, P., García-Gómez, J., Marcos, I. F. d., Rodrigo, M. A. & Lobato, J. Measurement of Mass-Transfer Coefficients by an Electrochemical Technique. *Journal of Chemical Education* **83**, 1204, doi:10.1021/ed083p1204 (2006).

6 Donaghue, A. & Chaplin, B. P. Effect of Select Organic Compounds on Perchlorate Formation at Boron-doped Diamond Film Anodes. *Environmental Science & Technology* **47**, 12391-12399, doi:10.1021/es4031672 (2013).
7 Crittenden, J. C., Trussell, R. R., Hand, D. W., Howe, K. J. & Tchobanoglous, G. *MWH's Water treatment : principles and design*. (John Wiley and Sons, 2012).
8 Pereira, L. A. M. *et al.* Diffusion Coefficients of Fluorinated Surfactants in Water: Experimental Results and Prediction by Computer Simulation. *Journal of Chemical & Engineering Data* **59**, 3151-3159, doi:10.1021/je500211w (2014).
9 Legrand, J., Dumont, E., Comiti, J. & Fayolle, F. Diffusion coefficients of ferricyanide ions in polymeric solutions — comparison of different experimental methods. *Electrochimica Acta* **45**, 1791-1803, doi:https://doi.org/10.1016/S0013-4686(99)00391-6 (2000).
10 Valentine Richard, L. & Wang, H. C. A. Iron Oxide Surface Catalyzed Oxidation of Quinoline by Hydrogen Peroxide. *Journal of Environmental Engineering* **124**, 31-38, doi:10.1061/(ASCE)0733-9372(1998)124:1(31) (1998).
11 Wang, L., Lu, J., Li, L., Wang, Y. & Huang, Q. Effects of chloride on electrochemical degradation of perfluorooctanesulfonate by Magnéli phase Ti4O7 and boron doped diamond anodes. *Water Research* **170**, 115254, doi:https://doi.org/10.1016/j.watres.2019.115254 (2020).
12 Peng, J. *et al.* Bicarbonate enhanced removal of triclosan by copper(II) catalyzed Fenton-like reaction in aqueous solution. *Chemical Engineering Journal* **306**, 484-491, doi:https://doi.org/10.1016/j.cej.2016.07.088 (2016).
